# Trend in age at menarche and its association with body weight, body mass index and non-communicable disease prevalence in Indonesia: evidence from the Indonesian Family Life Survey (IFLS)

**DOI:** 10.1186/s12889-022-12995-3

**Published:** 2022-03-31

**Authors:** Muhammad Asrullah, Monique L’Hoir, Edith J. M. Feskens, Alida Melse-Boonstra

**Affiliations:** Division of Human Nutrition and Health, Wageningen University and Research, Stippeneng 4, 6708 WE Wageningen, the Netherlands

**Keywords:** Menarche, Nutritional status, Non-communicable diseases, Indonesia

## Abstract

**Background:**

In western countries, age at menarche (AAM) is nowadays lower than a century ago, coinciding with increased Body Mass Index (BMI) and prevalence of non-communicable diseases (NCD). This study aimed to determine the time trend in AAM, and its association with BMI and NCD prevalence at later age, in Indonesia.

**Methods:**

We used secondary data of 15,744 women aged 15–65 years from the Indonesian Family Life Survey (IFLS) conducted in the period 1993 to 2015. Multiple linear regression was applied to determine the association of AAM with BMI, and Poisson regression with robust variance for investigating the association of AAM with NCD prevalence ratios. Models were adjusted for age, and effect modification by wealth status, living area, and region was investigated.

**Results:**

AAM has significantly declined from 14.4 (SD:2.1) years of age in the 1940s to 13.4 y (SD:1.5) in the 1990s. AAM was inversely associated with BMI (β: − 0.30 kg/m^2^, 95%CI: − 0.37, − 0.22) and body weight (β: − 0.67 kg, 95%CI: − 0.75, − 0.54), but was not associated with height. After adjustment for age, AAM was not associated with NCD, i.e. hypertension, type 2 diabetes mellitus, liver diseases, asthma, chronic lung diseases, cardiovascular diseases, stroke, cancer, or arthritis. Including BMI in the models did not change the results.

**Conclusions:**

From the 1940s to 1990s, AAM has declined with 1 year in Indonesia. Women with earlier AAM had higher BMI and body weight at later age, but AAM was not associated with NCD prevalence in later life in the Indonesian population. Further longitudinal research is needed to disentangle the direction of causality of the associations.

**Supplementary Information:**

The online version contains supplementary material available at 10.1186/s12889-022-12995-3.

## Introduction

In recent decades, non-communicable diseases (NCD) have emerged as the leading cause of death in both developing and developed countries. In Indonesia, six of the top 10 leading causes of death consisted of NCD in 2017, of which stroke was the number one cause from 2007 to 2017 [1]. Based on Indonesian National Health survey data, the prevalence of NCD increased significantly from 2013 to 2018. For example, the prevalence of stroke among people aged 15 years or older increased from 7.0 to 10.9%, type 2 diabetes mellitus (DM) from 6.9 to 8.5%, and hypertension from 25.8 to 34.1%. The prevalence of cancer, arthritis, and cardiovascular diseases (CVD) also increased, especially among the older age groups (> 55 years).

The etiology of NCD is explained by a multitude of factors such as ethnicity, age, physical activity, dietary patterns, smoking status, as well as early biological maturation [2]. Overweight and obesity, which have globally increased over the past fourty years [3], are known as the most important proximal risk factors for NCD [4]. Any increase in the prevalence of overweight and obesity should therefore be of concern. The prevalence of overweight, obesity and abdominal obesity among people aged 15 years or older in Indonesia increased from 8.6, 10.5 and 18.8% in 2007 to 13.6, 21.8, and 31.0% in 2018, respectively [5]. Overweight and obesity during childhood have been associated with accelerated physical maturation [6]. Obese girls experience their menarche at an earlier age than normal [7], but it is not clear if higher BMI is either a cause or a consequence of earlier maturation, or both. In addition, some studies have reported that earlier age at menarche (AAM) is associated with increased risk of NCDs in later life, such as stroke, cardiovascular diseases [8], and diabetes [9]. Moreover, AAM has shown to be predictive for cardiovascular disease events and mortality [8].

In Indonesia, a previous study showed that higher Body Mass Index (BMI), higher parental income, and living in an urban area were associated with earlier puberty in both girls and boys based on Tanner scale assessment (pubic hair, female breast development, and male external genitalia) [10]. Globally, menarche occurs at an earlier age than in previous decades [11, 12]. For example, in Mexico mean AAM decreased from 13.6 to 12.6 years of age from 1900s to 1980s, while undergoing rapid economic transition, and earlier AAM was associated with diabetes and hypercholesterolaemia [13]. Therefore, early AAM may be an intermediary factor between childhood obesity and the development of NCD.

We aimed to describe the time trend in AAM, and to identify any associations of AAM with obesity and NCD among women from diverse socio-economic groups, living areas, and regions in Indonesia by analysing nationally representative data from the Indonesian Family Life Survey. We expected these associations to be inverse.

## Material and methods

### Indonesian family life survey

#### Data collection

The Indonesian Family Life Survey (IFLS) has so far been conducted in 5 waves, namely in 1993, 1997–1998, 2000, 2007–2008, and 2014–2015. IFLS is the only large longitudinal survey in Indonesia and represents 83% of the Indonesian population. Administration of questionnaires and health measurements, such as anthropometry and blood analysis, has been conducted under supervision of the non-profit RAND Corporation (Santa Monica, CA, USA). The aim of the IFLS survey is to provide multi-factorial data on economic and non-economic behavioural variables and outcomes such as food consumption, health status, and insurance utilization.

The first wave of IFLS included 13 out of 27 provinces and included 22,000 individuals living in 7244 households, using a stratified random sampling technique. The sampling method considered the heterogeneity of the population and represents four out of the five largest islands of Indonesia; Sumatra, Java, Kalimantan, and Sulawesi. Data collection areas were adapted from the areas covered by the National Socioeconomic Survey (SUSENAS) in 1993, which was based on the 1990 census. The details of the sampling frame have been described previously in an online IFLS report [14].

IFLS wave 2 collected data from the same individuals as in 1997, covering approximately 94.4% of the first wave. The next IFLS data collection rounds (wave 3–5) mostly interviewed dynasty households, which means that they already participated in any of the previous IFLS data collection rounds. The proportion of dynasty data was 95.3% in 2000 (wave 3), 93.6% in 2007 (wave 4), and 92% in 2014 (wave 5). The proportion of successfully re-contacted households was higher for the IFLS than generally seen in surveys conducted in the United States and Europe. Data collection, comprising self-reported data and direct measurements, was conducted by duly trained data collectors.

#### Participants

**Fig. 1:**
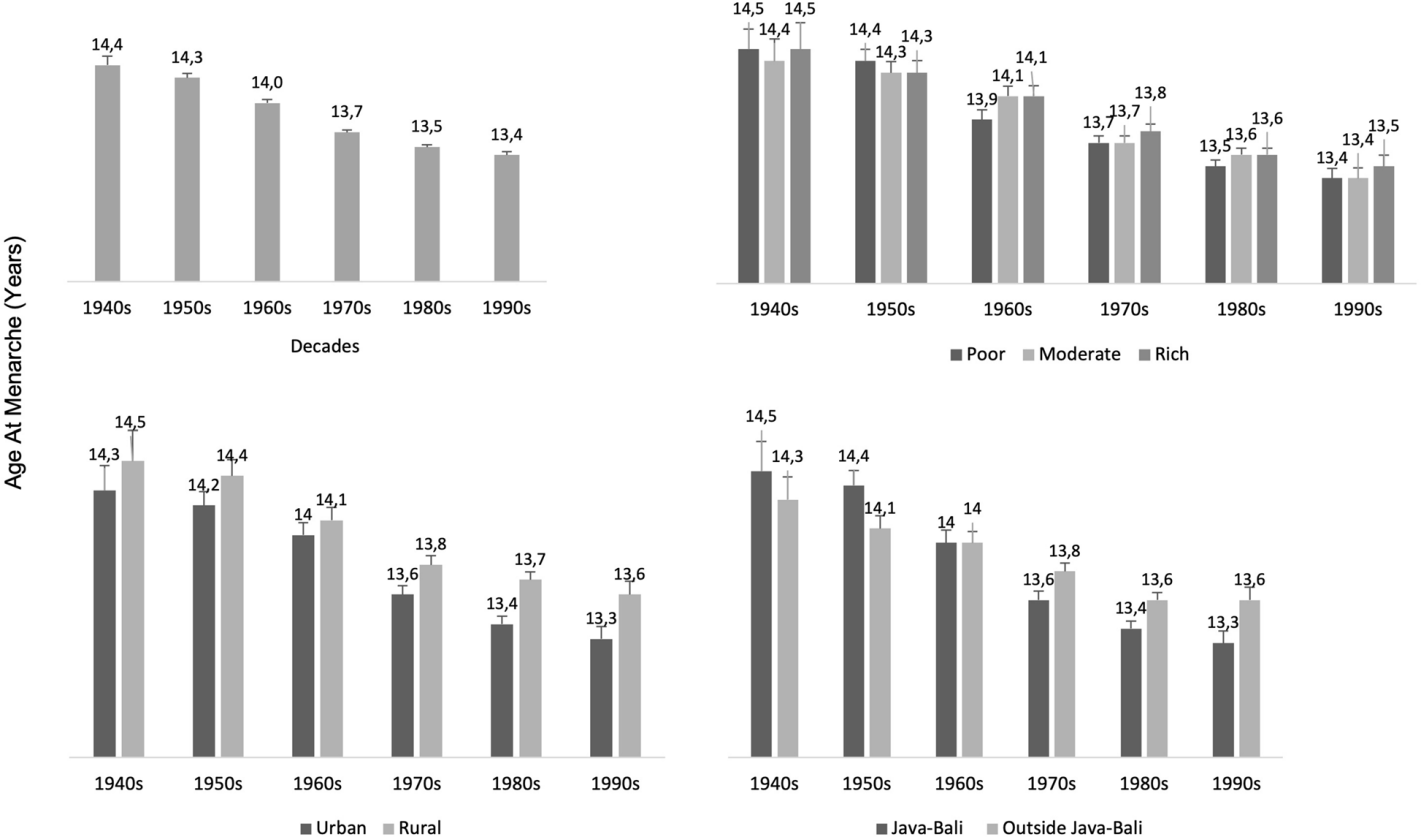
Time trend of AAM based on year of birth, wealth index, living area, and region among 15,744 respondents from IFLS waves 1–5

For the aim of the present analysis, data were selected of all women aged 15 years and older with a complete record for AAM, age, anthropometric indicators, and NCD from each IFLS wave. Information on AAM was only collected from married women, while NCD was assessed for all women, albeit only in wave 4 and 5. For the purpose of this study, only data of the most recent survey were included for women participating in multiple survey waves (Suplemental Fig. 1).

### Explanatory and outcome variables

AAM was assessed using a questionnaire by asking: “*how old were you (in years) at your first menstruation?*” We classified early AAM as < 12 years of age, normative AAM as in the range ≥ 12 – < 16 years, and late AAM as ≥16 years. This classification was based on the AAM distribution in our population, defined as plus and minus one standard deviation of the mean (mean: 13.75 and SD: 1.80). Similar age classification of AAM has been used in previous studies [15, 16].

Trained data collectors measured body weight of each participant using a body weight scale up to the nearest tenth of a kilogram or one single decimal, while height was measured using a Seca plastic height board (model 213) up to the nearest millimeter. BMI was calculated as weight (kg)/height (m)^2^ and classified into categories of normal weight (BMI < 25), overweight (BMI ≥25 - < 30) and obese (BMI ≥30).

Occurrence of NCD was assessed by questionnaire, asking whether or not participants had been diagnosed with any of the following chronic conditions: hypertension, type 2 diabetes mellitus, asthma, chronic lung disease, cardiovascular disease, liver disease, stroke, cancer and arthritis. Because not all waves collected these data, only wave 4 and 5 were included in the part of the analysis relating AAM to NCD.

Other variables that were included in our analysis were household socio-economic status, living area, and region. A variable for socio-economic status (SES) was constructed by assigning weights of eleven household assets (ownership of the house, ownership of another building, posession of farmland, fishpond or poultry, vehicles, electronic devices (e.g. radio, tv, refrigerator or washing machine), savings, jewellery, and other assets) into a five-quintile wealth index by Principle Component Analysis (PCA). For the present analysis, we categorized this index further into three groups; poor for the two bottom quintiles, average for the two middle quintiles, and rich for the highest quintile. The same classification of SES has been used for IFLS data in previous studies [17]. Participants were categorized into rural and urban areas of living using the classification of Indonesia’s Bureau of Statistics [18], which is based on population density, percentage of agricultural households, and presence/access to facilities.

### Statistical analysis

Data were analysed using STATA statistical software version 14. Data cleaning was employed at two levels; the first level was the data cleaning for AAM and BMI data which were extracted from all IFLS waves; and the second level was for NCD data, which were additionally extracted only from the last two waves (wave 4 and 5), since these were not available for the earlier waves. (Supplementary Fig. 1). Data on AAM were used to determine the time trend of AAM and its association with BMI, while NCD data were used to assess associations between AAM and NCD. Completeness of variables such as AAM, BMI, age, wealth index, and unplausible AAM and body weight because of input errors were considered in data cleaning. Wealth index, BMI and NCD were taken from the last wave in which the respondent was enrolled since these outcomes are influenced by age. Assumptions of normal data distribution was verified by visual inspection of Q-Q plots. In addition, multicollinearity was insepcted by plotting the correlation matrix of all independent variables and identifying the Variance Inflation Factor (VIF) for each variable. The correlation matrix showed that all correlation coefficientss were < 0.4, and the average VIF was < 2.

AAM, women’s weight, height, and BMI were described using means and standard deviations. We determined the mean AAM per decade, based on the birth year of participants, to investigate the time trend. Determinant variables in each wave were analyzed in relation to AAM by ANOVA and Chi-square test. Furthermore, associations between AAM and nutritional status (BMI, weight, and height) were determined by multiple linear regression and Poisson regression models with robust variance, adjusted for age. The data were stratified by wealth index, living area, and region, since these variables were found to be effect modifiers. Finally, we used Poisson regression models with robust variance to determine the association of AAM with the prevalence of NCD, while adjusting for age as well as for BMI as a potential intermediate.

## Results

### Characteristics of respondents

Characteristics of respondents are shown in Table 1. The total number of respondents was 15,744 women, and their average age was 36 years (SD:10.1 y). Most respondents were classified in the wealth index categories “poor” and “average”. More than half of respondents lived in urban areas and in the region of the islands Java and Bali.

**Table 1:** Characteristics of the selected female respondents according to IFLS wave (*N* = 15,744)

	IFLS 1 (*n* = 33)		IFLS 2 (*n* = 100)		IFLS 3(*n* = 1396)		IFLS 4(*n* = 2168)		IFLS 5(*n* = 12,047)		
	Mean	SD	Mean	SD	Mean	SD	Mean	SD	Mean	SD	*P*-value
Age, years	36.5	9.4	41.8	10.7	36.9	12.9	40.1	13.4	36.1	10.0	< 0.001
Age at Menarche, years	14.9	2.7	14.0	2.4	14.1	1.9	14.0	1.8	13.7	1.8	< 0.001
Nutritional status											
BMI, kg/m^2^	21.64	4.23	21.59	4.03	22.35	3.96	24.40	14.22	24.88	4.70	< 0.001
Weight, kg	49.7	9.9	47.4	9.5	50.5	9.8	53.4	11.4	57.0	11.52	< 0.001
Height, cm	151.5	6.1	148.0	6.1	150.2	5.3	149.9	8.9	151.4	5.5	< 0.001
	%		%		%		%		%		
Wealth Index, %											
Poor	39.4		26.0		33.4		35.2		42.1		< 0.001
Average	36.4		39.0		38.4		42.5		40.4		
Rich	24.2		35.0		28.2		22.3		17.5		
Living area, %											
Urban	36.4		29.0		45.9		51.8		57.3		< 0.001
Rural	63.6		71.0		54.1		48.2		42.7		
Region, %											
Java and Bali	60.6		64.0		62.7		61.2		55.1		< 0.001
Outside Java and Bali	39.4		36.0		37.3		38.8		44.9		

Note: ANOVA was used to analyse the age, Age at Menarche and nutritional status in each wave; Chi-square was applied for wealth index, living area, and region

### Time trend of AAM

AAM decreased from 14.4 (SD:2.1) years of age for women born in the 1940s to 13.4 (SD:1.5) years of age for women born in the 1990s. Mean AAM over the entire time period was 13.8 years (SD:1.8). Of all respondents, 5.8% were categorized as having early AAM (< 12 years), 81.6% as normative (≥12 – < 16 years), and 12.5% as late (≥16 years). AAM was not significantly different between women who lived in urban (mean:13.7 y, SD:1.7) and rural areas (mean:13.9 y, SD:1.8; p:0.08), who lived on the two main ilsands Java or Bali (mean:13.7 y, SD:1.9) or elsewhere (mean:13.8 y, SD:1.7; p:0.10), or who fell in different wealth status classifications; poor (mean:13.7 y, SD:1.8), average (mean:13.8 y, SD: 1.8), and rich, (mean:13.8 y, SD:1,9; p:0.10) (Fig. 1).

### AAM and body weight, height, and BMI

Table 2 shows the results of multiple linear regression models investigating the association between AAM and BMI. In unadjusted as well as in age-adjusted analysis, AAM (in years) was inversely associated with BMI (adjusted β: − 0.30 kg/m^2^ per year of AAM, 95%CI: − 0.37, − 0.22). When stratifying this analysis, the association appeared to be stronger in the highest wealth category (β: − 0.39 kg/m^2^ per year, 95%CI: − 0.43, − 0.26), in the urban area (β: − 0.35 kg/m^2^ per year, 95%CI: − 0.43, − 0.26) and at the two main islands (Java and Bali; β: − 0.33 kg/m^2^ per year, 95%CI: − 0.40, − 0.24) compared to the other socio-demographic categories. Further analysis using Poisson regression models adjusted for age showed that in later life, overweight and obesity were more prevalent among women with early age at menarche (overweight: PR: 1.11, 95%CI: 1.02–1.22; and obesity: PR: 1.35, 95%CI: 1.35–1.76) as compared to women with normative age at menarche, whereas the prevalence ratio of overweight and obesity among women with late age at menarche was lower (overweight: PR: 0.86, 95%CI: 0.81–0.94; and obesity: PR: 0.77, 95%CI: 0.67–0.87).

**Table 2:** Age-adjusted association between AAM and BMI at later age among female respondents of IFLS waves 1–5 (*N* = 15,744)

Variables		BMI (kg/m^**2**^)			
	*n* (%)	Unadjusted		Age-adjusted	
		β	(95% CI)	β	(95% CI)
Total population	15,744 (100)	−0.23	− 0.31, − 0.16	− 0.30	− 0.37, − 0.22
Wealth index^1^					
Poor	6345 (40.3)	− 0.21	− 0.31; − 0.11	− 0.29	− 0.39; − 0.19
Average	6371 (40.5)	−0.20	− 0.27; − 0.12	−0.24	− 0.31, − 0.17
Rich	3028 (19.2)	−0.33	− 0.49; − 0.17	−0.39	− 0.54; − 0.23
Living area^1^					
Urban	8705 (55.3)	−0.26	− 0.34; − 0.17	−0.33	− 0.41; − 0.25
Rural	7039 (44.7)	−0.17	− 0.25; − 0.08	−0.26	− 0.34 -0.17
Region^2^					
Java and Bali	8931 (56.7)	−0.25	−0.32; − 0.16	−0.33	− 0.40; − 0.24
Outside Java and Bali	6813 (43.3)	−0.22	− 0.30; − 0,13	−0.26	− 0.34; − 0.16

^1^*p*-value variable interaction: < 0.001

^2^*p*-value interaction: < 0.050

AAM was also inversely associated with current body weight (adjusted β: − 0.67 kg per year of AAM, 95%CI: − 0.75, − 0.54) (Table 3). The association was stronger in urban (β: − 0.68 kg per year of AAM, 95%CI: − 0.81, − 0.54) than in rural women (β: − 0.52 kg per year, 95%CI: − 0.67, − 0.38), whereas differences between other sociodemographic categories were small. The association between AAM and height was either small or absent (β: − 0.03 cm per year of AAM, 95%CI: − 0.09, 0.02). (Table 4).

**Table 3:** Age-adjusted association between AAM and body weight at later age among female respondents from IFLS waves 1–5 (N = 15,744)

Variables		Body Weight (kg)			
	*n* (%)	Unadjusted		Age-adjusted	
		β	(95% CI)	β	(95% CI)
AAM (years)	15,744 (100)	−0.58	− 0.68, − 0.48	−0.67	− 0.75, − 0.54
Wealth index^1^					
Poor	6345 (40.3)	−0.55	− 0.71; − 0.40	−0.67	− 0.83; − 0.50
Average	6371 (40.5)	−0.60	− 0.75; − 0.48	−0.67	− 0.83, − 0.51
Rich	3028 (19.2)	−0.53	− 0.74; − 0.32	−0.57	− 0.78 -0.35
Living area^1^					
Urban	8705 (55.3)	−0.54	−0.67; − 0.39	−0.68	− 0.81; − 0.54
Rural	7039 (44.7)	−0.50	− 0.64; − 0.35	−0.52	− 0.67; − 0.38
Region^2^					
Java and Bali	8931 (56.7)	−0.52	− 0.64; − 0.38	−0.61	− 0.74; − 0.48
Outside Java and Bali	6813 (43.3)	−0.67	− 0.82; − 0,50	−0.73	− 0.88; − 0.56

^1^*p*-value interaction: < 0.001

^2^*p*-value interaction: < 0.001

**Table 4:** Age-adjusted association between AAM and body height at later age among female respondents from IFLS waves 1–5 (*N* = 15,744)

Variables		Body Height (cm)			
	*n* (%)	Unadjusted		Age-adjusted	
		β	(95% CI)	β	(95% CI)
AAM (years)	15,744 (100)	−0.10	− 0.15, − 0.04	−0.03	− 0.09; 0.02
By wealth^1^					
Poor	6345 (40.3)	−0.09	−0.17; 0.01	− 0.01	−0.10; 0.07
Average	6371 (40.5)	−0.11	−0.20; − 0.04	−0.06	− 0.13, 0.03
Rich	3028 (19.2)	−0.06	− 0.18; − 0.06	−0.03	− 0.11; 0.12
By area^2^					
Urban	8705 (55.3)	−0.07	−0.14; < 0.01	< 0.01	−0.06; 0.08
Rural	7039 (44.7)	−0.09	−0.17; − 0.01	−0.02	− 0.10; 0.05
By region^3^					
Java and Bali	8931 (56.7)	−0.09*	−0.16; − 0.02	<−0.01	− 0.07; 0.06
Outside Java and Bali	6813 (43.3)	−0.10*	− 0.18; − 0,02	−0.06	− 0.14; 0.02

^1^*p*-value interaction: 0.911

^2^*p*-value interaction: 0.100

^3^*p*-value interaction: 0.330

### AAM and risk of NCD

Hypertension was the NCD with the highest prevalence (14.7%) while liver disease and stroke were less prevalent (0.8 and 0.6% respectively) in the study population. Although AAM was associated with an increased prevalence of hypertension, cardiovascular diseases and arthritis in crude models, these associations disappeared after adjusting for age and BMI (Table 5).

**Table 5:** Prevalence ratio of NCD outcomes with AAM (in years) among female respondents from IFLS wave 4 and 5 (*N* = 13,267)

Diseases Outcome	Frequency^a^	Crude model			Age-Adjusted			Age and BMI-adjusted		
	*n* (%)	PR	95% CI		PR	95% CI		PR	95% CI	
Hypertension	1950 (14.7)	1.03	1.01	1.06	0.98	0.96	1.01	0.99	0.97	1.01
Diabetes Mellitus	283 (2.2)	1.04	0.97	1.12	0.97	0.91	1.03	0.97	0.91	1.03
Asthma	399 (3.0)	1.02	0.96	1.08	1.03	0.97	1.1	1.03	0.97	1.1
Chronic lung diseases	197 (1.5)	0.98	0.90	1.06	0.97	0.90	1.05	0.95	0.88	1.03
Cardiovascular diseases	242 (1.8)	1.09	1.02	1.16	1.03	0.97	1.1	1.03	0.97	1.1
Liver diseases	99 (0.8)	1.08	0.97	1.2	1.08	0.99	1.22	1.09	1.001	1.23
Stroke	71 (0.6)	1.01	0.88	1.15	0.94	0.83	1.06	0.94	0.83	1.06
Cancer	134 (1.0)	0.97	0.89	1.05	0.96	0.88	1.04	0.96	0.89	1.04
Arthritis	826 (6.3)	1.1	1.06	1.14	1.02	0.99	1.06	1.02	0.99	1.06

Note: *PR* prevalence ratio. Independent variable, *AAM* dependent variable: NCD

^a^percentage was measured from total respondents

## Discussion

In this study, we found that among Indonesian women AAM declined over birth decades from 14.4 years in the 1940s to 13.4 years in the 1990s. Furthermore, AAM was inversely associated with BMI and body weight in later life, independently of age. AAM was also associated with a higher prevalence of several NCD such as hypertension, CVD, and arthritis, but this depended on age. Additional adjustment for BMI did not alter these results.

A previous study that reviewed published data indicated a similar time trend of AAM in Indonesia, with a decline from 14.4 y to 13.6 y since 1970 [19]. For China, also a decline in AAM has been reported, with an average of 16.2 y for women born before 1950 to 14.7 y for those born after 1959 [20]. Whereas AAM in China is estimated at 15.4 years for the period of 2004–2008, for Taiwan, it was 13.9 years for the same period of time; this disparity may have been influenced by the economic development in Taiwan since the mid-1980s, leading to changes in lifestyle and nutritional status, and thereby to earlier maturation of women [21]. Decline in AAM in both developed and developing countries has been associated with factors such as wealth index, access to health facilities, and intake of food, and it varies within populations based on the local context [22]. Nevertheless, AAM did neither differ between rural and urban areas, nor between different wealth groups in our study.

We observed a significant inverse association of AAM with BMI and body weight, indicating that each year of earlier menarche was associated with an increment in BMI of 0.30 kg m^− 2^, and of 0.67 kg in adult body weigth after adjustment for age. Previous research, both in western and Asian countries, showed a similar pattern [8, 23]. A systematic review showed that the mean difference in BMI of adult women with menarche < 12 years versus ≥12 years was 0.34 kg m^− 2^ [23]. This is less than what we found in the current study (1.13 kg m^− 2^) when using the same classification. In a survey among 12,336 Korean women it was reported that early AAM (defined as ≤11 years) was correlated with a higher BMI [24]. The same trend was also seen in a number of longitudinal studies from the UK that made use of mendelian randomization by genetic traits related to pubertal onset, although in one of these cohorts the association attenuated after adjusting for pre-pubertal BMI [25, 26].

The question arises whether increased body mass predisposes girls to earlier puberty, or that earlier puberty in physical and hormonal changes contributes to increased body (fat) mass in later life. In support of the prior pathway, a longitudinal study showed that girls with a higher percentage of body fat at 5 years of age, and higher BMI and waist circumference at 7 years of age, were more likely to be classified with earlier pubertal development at 9 years of age. Earlier pubertal development was assessed by breast development, level of estradiol, and pubertal developmental scale (PDS) [27]. Also, a higher BMI-for-age z score at 9 and 43 months of age, and rapid increase in BMI during childhood, were associated with having menarche before 12 years of age among 2083 women in Southern Brazil [28]. Furthermore, fast increases in BMI at 4 months, 1 year, and 4 year of age were associated with increased likelihood of having a 4.6-month earlier menarche in the United States [29]. This may be explained by the fact that children with higher BMI are exposed to higher circulating concentrations of leptin, which could stimulate gonadotropin releasing hormone and premature secretion of sex hormones [30]. A 4-year longitudinal study among adolescents showed that AAM indeed declined by approximately 1 month per 1-ng/mL increase in leptin concentration [31]. In contrast, however, cohort data from India, Peru, and Vietnam consistently showed that increases in BMI during childhood (1–8 years of age) were not associated with earlier menarche [32]. The discrepancy between findings might be explained by the lower obesity prevalence in low- and middle-income countries (LMICs) compared to advanced economies such as the United States and some Latin American and European countries [33, 34]. Despite rapid increases in the prevalence of childhood overweight in LMICs, the prevalence of undernutrition also remains high [32].

There is also evidence that early maturation may induce higher BMI in later life [35]. Prospective cohort studies conducted in the UK and in Australia showed that earlier menarche was related to higher BMI in middle age, independent of their BMI at 4–6 years of age, and at 20 years of age, independent of their BMI at 8 years of age, respectively [36, 37]. Furthermore, a systematic review on genetic predisposition of pubertal timing by mendelian randomization found that earlier pubertal timing assessed by several pubertal traits such as age at menarche, age at voice breaking, male genital and female breast development, was related to increased BMI at adult age [38]. Therefore, both pathways may play a role in the relationship between AAM and BMI which cannot be disentangled by our cross-sectional data.

Although we did not find any association with height, a previous large prospective study with data from 286,205 women from nine European countries found that they were 0.31 cm taller for each year of later menarche [39]. A prospective study in South Africa also showed an increase in adult height of 1.15 cm per year of later menarche after adjusting for prepubertal height and BMI [40]. It has been hypothesized that women with early menarche experience premature closure of the epiphyseal growth plate which is responsible for elongation of the bones. Therefore, later menarche would allow further development of the long bones resulting in a greater adult height [39].

Regarding NCD, a number of large studies did find associations between AAM and cardiovascular diseases, hypertension, and stroke, both in developed and developing countries [8, 20, 24], whereas some others, consistent with our findings, did not [13, 41]. Among 118,964 women with breast cancer from 117 epidemiological studies, it was shown that with every year decline in AAM, the risk for breast cancer increased with 5% [42]. Early menarche (< 12 years) was also identified to increase the risk of asthma [43]. Furthermore, a one-year earlier menarche was associated with a higher risk of Non-Alcoholic Fatty Acid Disease (NAFLD) [9, 44]. The effect of AAM on rheumatoid arthritis is still under debate, with some studies showing a protective effect for early AAM [45], as well as for late AAM [46], whereas others reported no association [41]. The longer estrogen exposure among women with earlier AAM may provide a possible mechanism for the related higher risk of NCD [39]. In addition, the higher BMI in middle age seen with earlier AAM may mediate the association with NCD. The prevalence of obesity among Indonesian women has increased while AAM has declined, as shown in this study. Even though the prevalence of NCD has also risen, it may not be at its highest point yet. The incidence of high blood pressure, type 2 diabetes mellitus and stroke is still much lower in Indonesia than for example in the UK [8], China [20], and Korea [24]. This may explain why an association between AAM and NCD for Indonesia could not yet be shown, although an association between AAM and BMI has been clearly demonstrated. More studies with large sample sizes and small confidence intervals are needed in similar populations to clarify the relationship between AAM and NCD.

This is the first study using nationally representative data to explore the trend of AAM and its association with nutritional status and disease outcomes in Indonesia. The longitudinal nature of this study allowed us to assess the trend in AAM over six decades based on the year of birth. This study highlights an inverse association between AAM and BMI, with the latter known to be the most important proximal risk factor for NCD. There are some limitations to our study. For instance, the retrospective recall of NCDs or AAM might not be accurate, although the respondents of IFLS were generally healthy and able to remember their health history. Self-reported AAM collected by personal interview, like we used for this study, was shown to be consistent with AAM recall 30 years earlier in a retrospective follow-up study [47], as well as in a study with repeated interviews of menstrual history in the period of 1985–1993 [48]. To better understand the associations between AAM, BMI and NCD, prospective, longitudinal research with more details on pubertal stage and measurement of sex hormones and other biological factors are needed. Full characterization of body compisition would also be needed instead of relying on BMI alone [35]. In the current study, no data on prepubertal BMI was available from the women included in the first two waves, nor for NCD data in the first three waves of IFLS. Possible selection bias may have occurred since data on AAM was only collected from married women. The analysis may have selectively excluded women due to early mortality or non-response; nevertheless, the number of missing cases was relatively small and therefore we do not expect it would change our conclusion.

## Conclusion

In Indonesia, AAM declined over birth decades from 14.4 years in the 1940s to 13.4 years in the 1990s. We found that women from the Indonesian Family Life Survey with earlier AAM had a higher BMI and body weight later in life. Nevertheless, AAM was not independently associated with any of the NCD outcomes. With the prospect of rising overweight and obesity prevalence, future longitudinal cohort studies should include pubertal stage with comprehensive indicators, and including all risk factors related to early AAM, to unravel causal pathways.

## Supplementary Information

**Additional file 1.**

## Data Availability

The data are available at https://www.rand.org/well-being/social-and-behavioral-policy/data/FLS/IFLS.html

## Abbreviations

AAM: Age At Menarche
BMI: Boddy Mass Index
NCD: Non-Communicable Diseases
SD: Standard Deviation
IFLS: Indonesian Family Life Survey
SUSENAS: The Indonesia National Socio-economic Survey
PCA: Principal Component Analysis
SES: Socio-economic Status
LMIC: Low-and-Middle Income Countries
NAFLD: Non-alcoholic Fatty Acid Disease
