# Supplementary figures and images for: Trend in age at menarche and its association with body weight, body mass index and non-communicable disease prevalence in Indonesia: evidence from the Indonesian Family Life Survey (IFLS)
Source: BMC Public Health. 2022 Mar 31;22:628. doi: 10.1186/s12889-022-12995-3 (PMC8969286; doi:10.1186/s12889-022-12995-3)

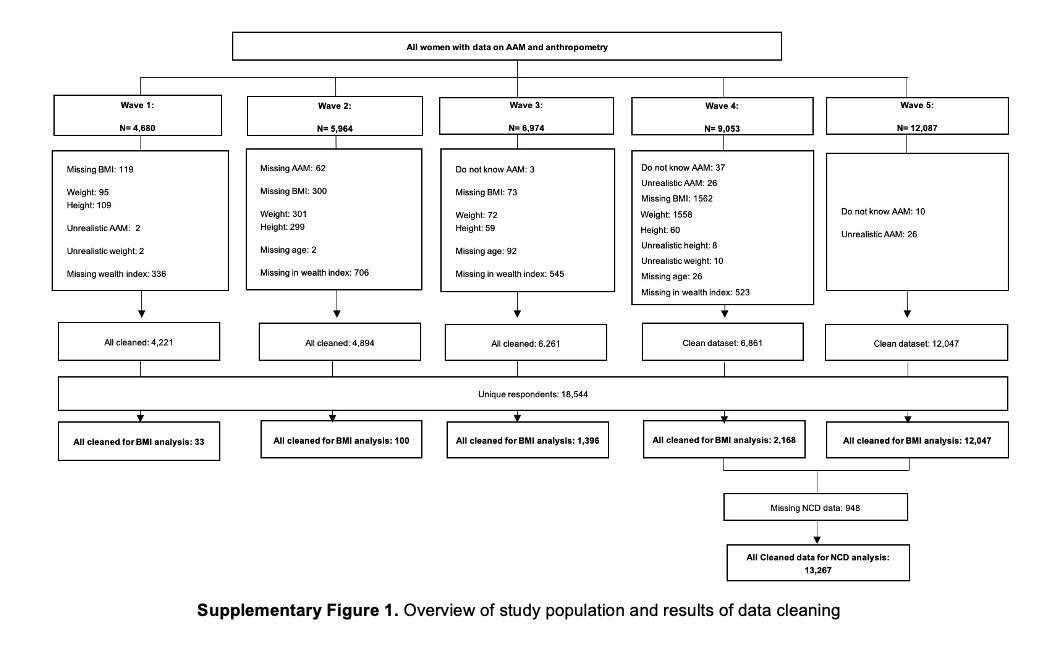

Supplement: Supplementary file 1 — Additional file 1. [file 12889_2022_12995_MOESM1_ESM.jpg]
